# A Novel Antibiotic Mechanism of l-Cyclopropylalanine Blocking the Biosynthetic Pathway of Essential Amino Acid l-Leucine

**DOI:** 10.3390/molecules22122224

**Published:** 2017-12-14

**Authors:** Bingji Ma, Jinwen Shen, Wandee Yindeeyoungyeon, Yuan Ruan

**Affiliations:** 1 Department of Traditional Chinese Medicine, Henan Agricultural University, Zhengzhou 450002, China; ruanyuanmbj@163.com; 2 Department of Microbiology, Henan Agricultural University, Zhengzhou 450002, China; shenjinwen369@163.com; 3 National Center for Genetic Engineering and Biotechnology, NSTDA, Pathum Thani 12120, Thailand; wandee@biotec.or.th

**Keywords:** l-cyclopropylalanine, anti-fungal activity, α-isopropylmalate synthase, natural antibiotics

## Abstract

The unusual amino acid l-cyclopropylalanine was isolated from the mushroom *Amanita virgineoides* after detection in an anti-fungal screening test. l-Cyclopropylalanine was found to exhibit broad-spectrum inhibition against fungi and bacteria. The anti-fungal activity was found to be abolished in the presence of the amino acid l-leucine, but not any other amino acids, indicating that l-cyclopropylalanine may block the biosynthesis of the essential amino acid l-leucine, thereby inhibiting fungal and bacteria growth. Further biochemical studies found l-cyclopropylalanine indeed inhibits α-isopropylmalate synthase (α-IMPS), the enzyme that catalyzes the rate-limiting step in the biosynthetic pathway of l-leucine. Inhibition of essential l-leucine synthesis in fungal and bacteria organisms, a pathway absent in host organisms such as humans, may represent a novel antibiotic mechanism to counter the ever-increasing problem of drug resistance to existing antibiotics.

## 1. Introduction

The excessive use of broad-spectrum antibiotics and other uncontrolled non-medical applications have resulted in a rapid onset of antibiotic resistance [1]. To overcome this resistance, antibiotic candidates or leads that act at new targets or via unprecedented mechanisms have the greatest potential. It should be emphasized that the main classes of antibiotics currently used originated from natural products and were discovered over 50 years ago, largely by classic screening techniques [2]. The prevalence of natural products and the derivatives thereof, may be due to their structural diversity, functional complexity and unique molecular architectures [3].

**Figure 1:**
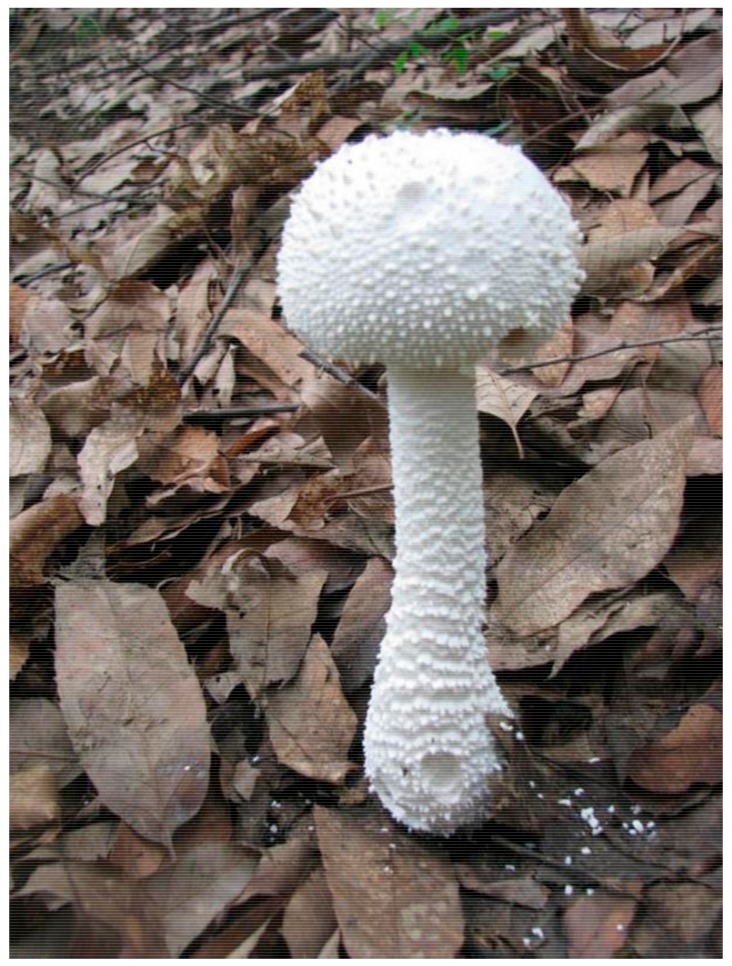
The mushroom *Amanita virgineoides* (the sample was collected in July 2010 by Bingji Ma and Jinwen Shen from Funiu Mountain in Henan Province, China).

**Figure 2:**
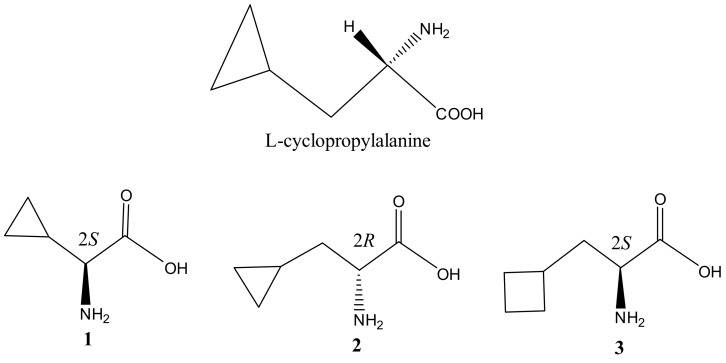
l-Cyclopropylalanine and its derivatives compounds **1**, **2** and **3**.

As a part of our ongoing work on seeking active anti-fungal metabolites from wild mushrooms, an unusual amino acid, l-cyclopropylalanine, was isolated from mushroom *Amanita virgineoides* (Figure 1 and Figure 2). An interesting phenomenon was observed in the screening test of l-cyclopropylalanine on potato dextrose agar (PDA) plates. The toxicity of this amino acid to fungi can be reversed in each case by addition of l-leucine to the PDA medium but not l-isoleucine or l-valine. In a view of high structural similarity between l-cyclopropylalanine and l-leucine, this experimental phenomenon suggests that the anti-fungal mechanism is either by displacing l-leucine directly in the synthesis of proteins or it is somehow involved in l-leucine metabolism. The animal toxicity test excluded the first possible antibiotic mechanism and as a result, l-cyclopropylalanine was found to be an α-isopropylmalate synthase (α-IMPS) inhibitor via mimicking feedback inhibition, thus blocking the biosynthetic pathway of l-leucine. This new antibiotic mechanism might be helpful to overcome the pressing problem of drug resistance to existing antibiotics.

## 2. Results and Discussion

### 2.1. Anti-Fungal Activity of l-Cyclopropylalanine and Its Derivatives

**Figure 3:**
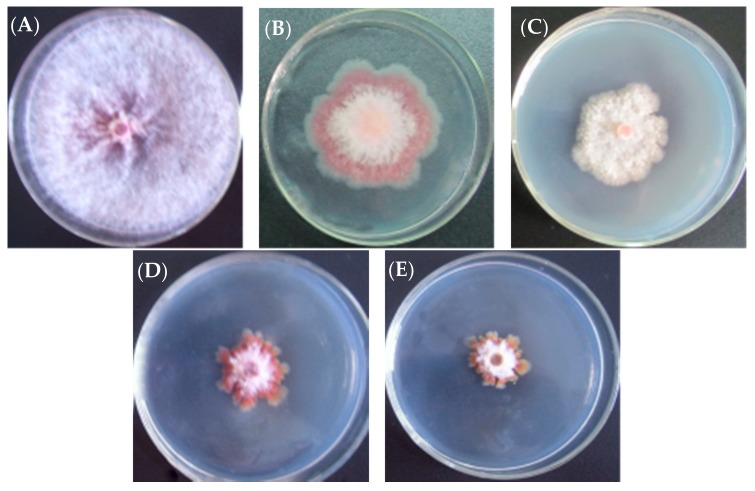
Inhibitory effects of l-cyclopropylalanine on *F. graminearum* Schw on PDA plates at different concentrations: (**A**) 0 μg/mL; (**B**) 3.13 μg/mL; (**C**) 6.25 μg/mL; (**D**) 12.5 μg/mL; (**E**) 25 μg/mL).

l-Cyclopropylalanine exhibited significant anti-fungal activity against twelve pathogenic fungi on PDA plates (Figure 3, Table 1). In addition, this unusual amino acid was previously reported to inhibit the spore germination of *Pyricularia oryzae* [4]. This anti-fungal activity is unique in that it shows little if any toxicity in animal tests. Rats were fed by gavage with l-cyclopropylalanine at a maximum dosage of 6 g/kg for 6 days. All rats appeared to be normal as control rats. Thus, the observed toxicity of l-cyclopropylalanine is likely selective towards fungal organisms.

To begin to understand the inhibitory effect of l-cyclopropylalanine against fungi, three derivatives of this unusual amino acid, namely compounds **1**, **2** and **3** were applied to an anti-fungal test using the same method (Figure 2). Comparing to l-cyclopropylalanine, compound **1** showed a strong inhibitory activity but at a higher concentration against *Fusarium graminearum* Schw, compounds **2** and **3** only displayed weak inhibitory effect (Supplementary Materials, Table S1). The results indicated that the β-cyclopropyl group and the 2*S* configuration of l-cyclopropylalanine were necessary and critical for its anti-fungal activity.

### 2.2. Anti-Fungal Effect of l-Cyclopropylalanine Reversed by l-Leucine

**Figure 4:**
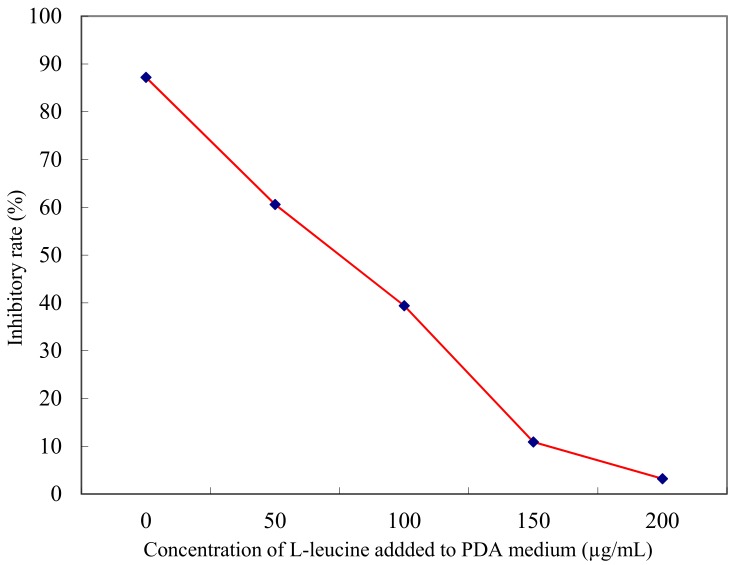
Anti-fungal effect of l-cyclopropylalanine reversed by l-leucine.

Considering that structurally l-cyclopropylalanine could be viewed as a product of cyclization of the two –CH_3_ in the l-leucine side group, it was speculated that the anti-fungal activity of l-cyclopropylalanine might be related to l-leucine, an essential amino acid. We therefore tested if l-leucine affects the anti-fungal activity of l-cyclopropylalanine. As shown in Figure 4, the inhibition rate of l-cyclopropylalanine against *F. graminearum* Schw decreases markedly with the addition of l-leucine. Equally important, addition of either l-isoleucine or l-valine or any other amino acids did not abolish the anti-fungal activity of l-cyclopropylalanine (data not shown). Taken together, these results indicate that l-cyclopropylalanine inhibits fungal growth likely by blocking the biosynthesis of l-leucine, an essential amino acid for growth.

### 2.3. Inhibition of α-IPMS by l-Leucine and l-Cyclopropylalanine

A key step in the l-leucine synthetic pathway is catalyzed by enzyme α-isopropylmalate synthase (α-IPMS), which converts the intermediate α-ketoisovalerate to α-isopropylmalate. It is known that α-IPMS is a highly conserved enzyme and inhibited by the l-leucine via a feedback mechanism [5,6,7,8]. Based on the effect of l-leucine on the anti-fungal activity of l-cyclopropylalanine and the structural relatedness between l-leucine and l-cyclopropylalanine, we suspected that l-cyclopropylalanine may also inhibit α-IPMS.

**Figure 5:**
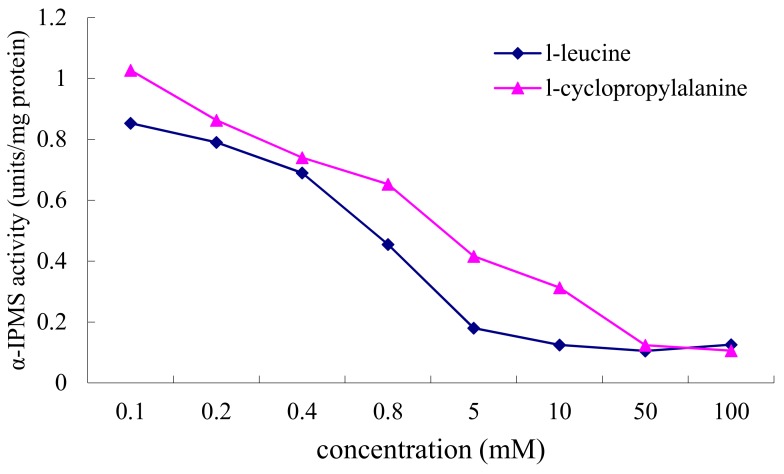
Inhibition of α-IPMS by l-leucine and l-cyclopropylalanine.

To test above hypothesis, α-IPMS from *M. tuberculosis* was cloned and expressed in *E. coli* BL21 as previously reported [9,10,11]. The enzyme was expressed in a good yield with an expected size of 74 kDa on SDS-PAGE. Using purified enzyme, inhibition assays of α-IPMS by l-leucine and l-cyclopropylalanine were performed, respectively. Enzymatic properties showed the purified α-IPMS worked well at 37 °C, pH 8.5. The inhibition test was conducted with the addition of 0.1–100.0 mM of l-leucine and l-cyclopropylalanine to the enzyme assay mixtures, respectively. The results showed that the inhibition effect of l-leucine is detectable at 0.4 mM l-leucine, and the enzyme was inhibited significantly at concentrations of greater than 0.8 mM (Figure 5). l-Cyclopropylalanine showed similarly inhibitory activity, but at a higher concentration. The α-IPMS was inhibited 50% in the presence of about 4 mM l-cyclopropylalanine. Furthermore, both l-leucine and l-cyclopropylalanine exhibited almost the same inhibitory activity on the enzyme once the concentration exceeded 50 mM. These biochemical data show that l-cyclopropylalanine indeed inhibits α-IPMS as l-leucine does.

### 2.4. Inhibitory Effect of l-Cyclopropylalanine against S. cererisiae, C. albicans and E. coli

Fungi, bacteria and plants contain biosynthetic pathway to produce l-leucine, an essential amino acid for growth. The biosynthetic pathways are highly conserved among these organisms. We next tested if l-cyclopropylalanine inhibits the growth of the more widely known fungus *Saccharomyces cererisiae* and the infectious fungus *Candida albicans* and bacterium *E. coli*.

**Figure 6:**
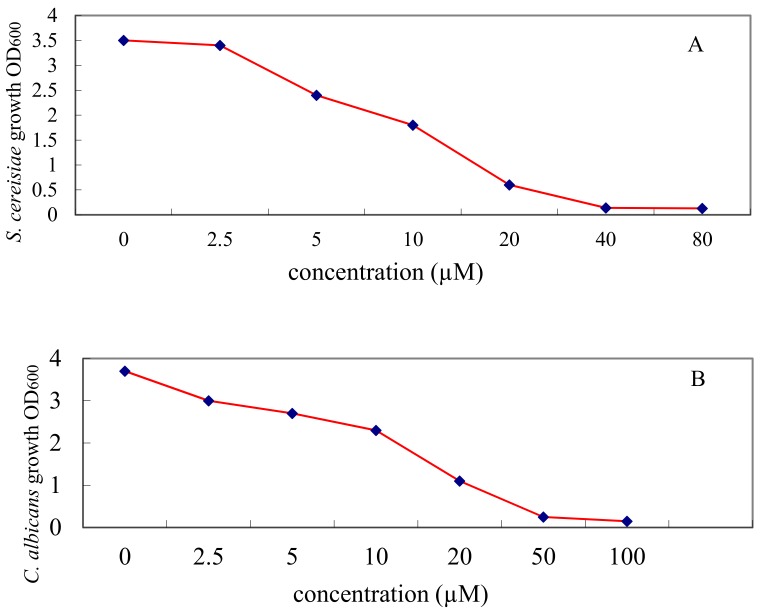

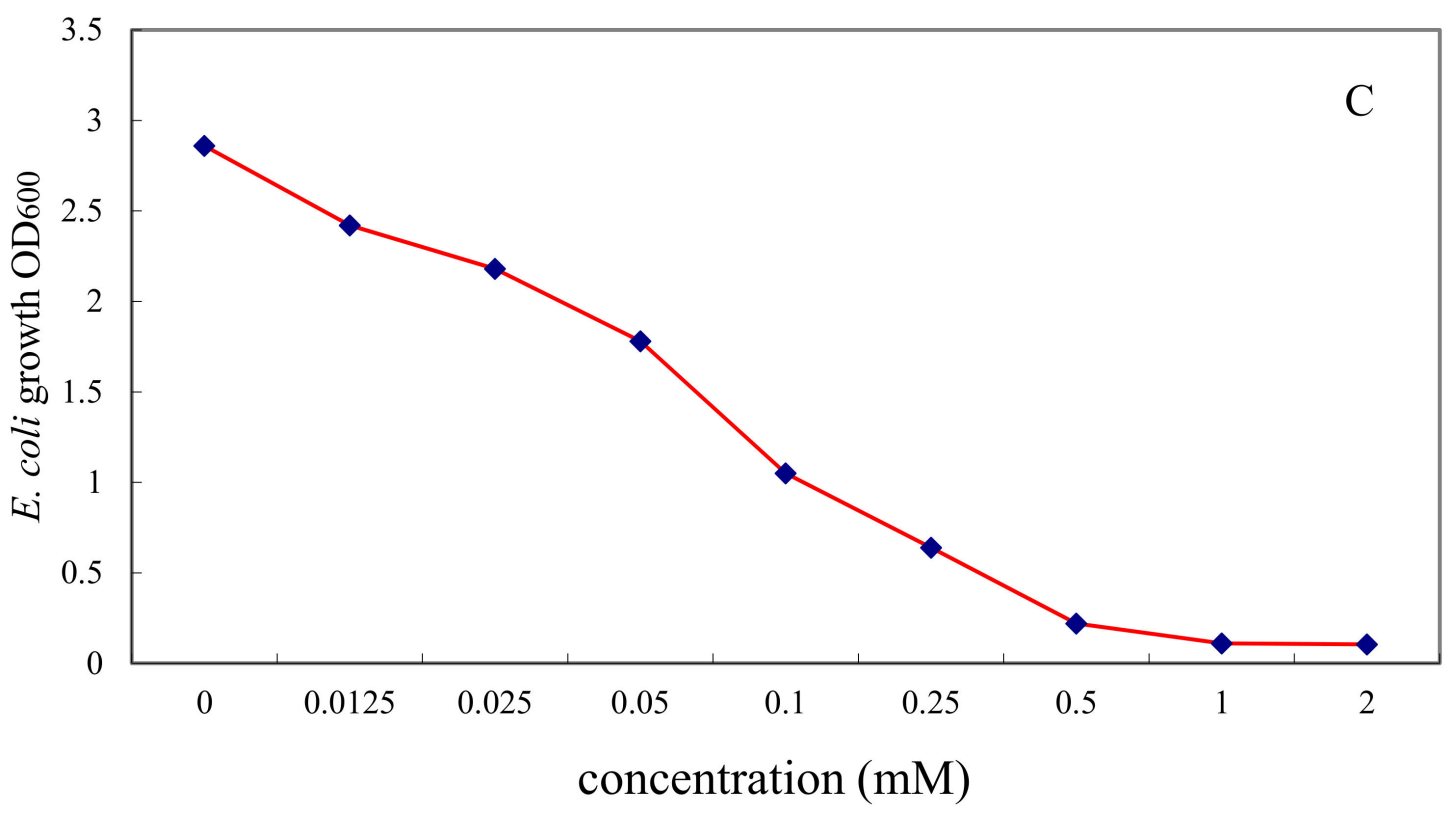
Inhibitory effects of l-cyclopropylalanine against *S. cererisiae* (**A**); *C. albicans* (**B**) and *E. coli* (**C**).

To eliminate l-leucine in the culture medium, an SD-leucine medium was prepared to culture the test fungi. As shown in Figure 6A,B, l-cyclopropylalanine showed a strong inhibitory effect on *S. cererisiae* and *C. albicans* with a minimum inhibitory concentration (MIC) of 19.2 µM and 44.5 µM, respectively. In the case of *E. coli*, M9 culture medium was selected for growing the test bacterial to exclude l-leucine in the medium. l-Cyclopropylalanine showed a less strong activity with a MIC 0.48 mM (Figure 6C). On the contrary, l-cyclopropylalanine only showed weak toxicity to *E. coli* once Luria-Bertani (LB), which is rich in l-leucine, was used to replace the M9 medium in the test. The results show that l-cyclopropylalanine has a broad anti-fungal activity and moderate anti-bacteria activity.

### 2.5. Discussion

When the new screening techniques failed to deliver suitable leads for drug candidates, interest in natural products flared up again [12]. Besides the conventional resources, some new natural resources, such as ocean microbes and endophytic fungi were explored to find the new antibiotics [13]. In this paper, we firstly reported the anti-fungal activity of l-cyclopropylalanine and its unexpected antibiotic mechanism. Mammals such as humans are not equipped to synthesize l-leucine, thus, l-leucine is acquired through protein-rich foods. In contrast, fungi, bacteria and plants contain biosynthetic pathway to produce l-leucine, an essential amino acid for growth. Hence, chemicals blocking the biosynthesis of essential amino acids will have the potential to develop as a new antibiotic. Consistent with our hypothesis, one of the other non-protein amino acid, 2-amino-3-cyclopropylbutanoic acid was reported to inhibit bacterial growth and this anti-bacterial activity was abolished by isoleucine, but not by leucine or valine [14]. It is known that α-IPMS is a highly conserved enzyme among fungi and bacteria including tuberculosis-causing *M. tuberculosis* and gonorrhea-causing *Neisseria gonorrhoea*. By blocking the biosynthetic pathway absent in the host organisms but present and essential for fungal and bacteria growth may circumvent current antibiotics problems such drug resistance and toxicity. Therefore, l-cyclopropylalanine may represent a valuable lead compound for the development of new antibiotics.

## 3. Materials and Methods

### 3.1. Chemicals and Materials

Acetyl CoA, CoA, 5,5′-dithiobis(2-nitrobenzoic acid) (DTNB), α-ketoisovaleric acid, carbendazim (*N*-1*H*-benzimidazol-2-yl-methyl ester, C_9_H_9_N_3_O_2_) and l-leucine were obtained from Sigma-Aldrich (Saint Louis, MO, USA). Three derivatives of l-cyclopropylalanine, compounds **1**, **2** and **3**, were purchased from Hanhong Biochemicals (Xuhui, Shanghai, China). All other chemicals were obtained from commercial sources and were of reagent grade. In the whole-cell inhibition assays, M9 (MgSO_4_, CaCl_2_, Na_2_PO_4_, KH_2_PO_4_, NaCl, NH_4_Cl and glucose as the components) and SD-leucine culture media were selected for growing *Escherichia coli* and *Saccharomyces cererisiae*, respectively. Restriction enzymes and T4 DNA ligase were obtained from New England Biolabs (Beverly, MA, USA). *Taq* DNA polymerase was obtained from Invitrogen (Carlsbad, CA, USA). *E. coli* strain DH5α was used for maintaining and cloning plasmid DNA. *E. coli* BL21 (λDE3) was used for protein expression. Recombinant plasmid containing *leuA* gene was used to express recombinant His_6_-α-IPMS. The fresh fruiting bodies of *A. virgineoides* were collected on Funiu Mountain of Henan Province, China, in July 2010. The mushroom identification was made by Prof. Jin-Wen Shen, Henan Agricultural University. A dried specimen was deposited in the Herbarium of Henan Agricultural University.

### 3.2. Anti-Fungal Test In Vitro and Isolation of the l-Cyclopropylalanine

Three hundred g air-dried fruiting bodies of *A. virgineoides* were extracted successively with ethyl acetate (1000 mL) and water (600 mL). Both the ethyl acetate and water extracts were tested for anti-fungal activity in vitro by the Poison Food Technique [15]. PDA medium was used as the medium for all test pathogenic fungi. The medium incorporating the ethyl acetate or water extract at concentration of 5 mg/mL was inoculated at the center with agar discs of test fungi (4 mm diameter). Three replicate plates for each fungus were included. After incubation for 4~6 days until the fungal growth in the control dishes was almost complete, the mycelial growth of fungi in both treated (*T*) and control (*C*) Petri dishes was measured diametrically in three different direction. The percentage of growth inhibition (*I*) was then calculated using the formula below:

*I* (%) = [(*C* − *T*)/*C*] × 100

The water extract in PDA plates showed strong inhibitory activity against the mycelial growth of plant pathogenic fungi. In order to isolate the active principle, the water extract (12.5 g) was fractionated by silica gel column chromatography and eluted by CHCl_3_/MeOH (9:1, 8:2 and 7:3, *v*/*v*) to give three fractions. The fraction (0.8 g, eluted by CHCl_3_/MeOH 7:3, *v*/*v*) was confirmed to be the active fraction by mycelial growth inhibition test in vitro, with the growth inhibition 73.2% at 1 mg/mL. Then the active fraction was submitted for further purification by recrystallization to give a pure compound (90 mg), which was identified as previously reported l-cyclopropylalanine [5] (Figure 2 and 1D-NMR, 2D-NMR and MS spectral in Supplementary Materials Figure S1).

### 3.3. Animals

Sprague-Dawley (SD) rats, weighing 200–250 g, were purchased from the Experimental Animal Center of Zhengzhou University. All the animals were housed in polypropylene plastic cages at room temperature with a relative humidity of 55–60%, and fasted for 18 h prior to the experiments, but provided with tap water ad libitum. Animal experiment was performed in accordance with institutional guidelines for Laboratory Animal Care of Experimental Animal Center, Henan Agricultural University. The Departmental Animal Ethical Committee of the Henan Agricultural University approved this study (HAU20150412).

### 3.4. Cloning of the leuA Gene by PCR Amplification

α-IMPS encodes as *leuA* and the primers of *leuA* gene from *Mycobacterium tuberculosis* strain H37Rv containing two copies of Variable Number of Tandem Repeats (VNTR) were designed for PCR amplification. Forward: 5′-GGAATTCCATATGACAACTTCTGAATCGCCC-3′ (*Nde* I); Reverse: 5′-CGCGGATCCCTAGCGTGCCGCCCGGTTGAC-3′ (*BamH* I). PCR products were purified using a PCR purification kit and then ligated to the vector pET15b. Correct clones were confirmed by restriction enzyme digestion and DNA sequencing.

### 3.5. Protein Expression and Purification

*E. coli* BL21 (λDE3) cells harboring recombinant plasmids were grown at 37 °C in LB medium supplemented with 100 μg/mL ampicillin until the culture reached mid log phase (0.3–0.4, OD_600_). Isopropyl-1-thio-β-d-galactoside (IPTG) was added to the culture to the final concentration of 0.5 mM. The culture was incubated at 20 °C and shaken for overnight. The bacterial cells were harvested and protein purification was carried out by Immobilized Metal ion Affinity Column (IMAC) method using Talon SuperFlow Metal Affinity Resin (Clontech, Mountain View, CA, USA) as recommended by the manufacturer. The protein was dialyzed (to remove immidazole) and concentrated using 3 kDa Amicon Ultra centrifugal filter (Millipore, Burlington, MA, USA), and kept in 1× equilibrium buffer (50 mM Sodium phosphate, 300 mM NaCl, pH 7.0) and 50% glycerol. The enzyme was expressed in a good performance with an expected size of 74 kDa on SDS-PAGE.

### 3.6. Enzyme Assay

The assays were performed to measure inhibition of the natural antibiotic l-cyclopropylalanine, compared to biological end-point inhibition of l-leucine for the enzyme α-IMPS. The assay used was an end-point assay, in which DTNB was used to detect formation of CoA at 412 nm. Reaction mixtures of 250 µL containing 50 µmol Tris-HCl, pH 8.5, 20 µmol KCl, 0.2 µmol acetyl CoA and 0.5 µmol α-ketoisovaleric acid. In the reaction mixtures, l-leucine and l-cyclopropylalanine were diluted in 50 mM Tris-HCl, pH 8.5. In the inhibition assays, both l-leucine and l-cyclopropylalanine were at a final concentration of 0.1, 0.2, 0.4, 0.8, 5.0, 10.0, 50.0 and 100.0 mM.

Reaction mixtures were pre-incubated at 37 °C for 5 min. Enzyme was subsequently added in a volume of 1 µL, and incubated at 37 °C for 5 min. The reaction was stopped with 0.75 mL absolute EtOH, and colored with 0.5 mL of 1 mM DTNB (DTNB diluted in 50 mM Tris-HCl). Reactions were analyzed using Thermo UV-visible spectrometer (Waltham, MA USA,) at OD_412_. A CoA standard curve was performed to measure relative concentrations of CoA formation. Additionally, one unit of enzyme is defined as the amount catalyzing the formation of 1 µmol CoA per minute. Enzyme activity is defined as units (of enzyme) per milligram protein.

## 4. Conclusions

In summary, l-cyclopropylalanine was isolated from the wide mushroom *A. virgineoides* and it exhibited a broad-spectrum inhibitory effect against fungi and bacteria. Biochemical studies demonstrate that l-cyclopropylalanine inhibits α-IPMS, a critical enzyme in the biosynthetic pathway of essential amino acid l-leucine. It is therefore possible that by inhibiting α-IPMS, l-cyclopropylalanine blocks the biosynthesis of essential amino acid l-leucine and in turn inhibits fungal growth, which represents a novel antibiotic mechanism.

## Figures and Tables

**Table 1:** Inhibitory effects of l-cyclopropylalanine on 12 plant pathogen fungi in PDA plates.

Pathogen Fungi	l-Cyclopropylalanine		Carbendazim **	
	IR * at 50 μg/mL	IR at 5 μg/mL	IR at 50 μg/mL	IR at 5 μg/mL
*Fusarium graminearum* Schw	77.2	65.5	22.7	8.6
*Fusarium moniliforme*	74.1	22.6	3.8	2.5
*Colletotrichum gloeosporioides* Penz	74.9	29.6	72.2	21.8
*Sclerotinia sclerotiorum* (Lib.) de Bary	100	20.8	100	8.0
*Ascochyta gossypii* Syd.	86.2	44.9	0	0
*Alternaria solani*	69.8	10.6	0	0
*Corynespora cassiicola*	69.0	42.0	0	0
*Colletotrichum capsici*	62.1	24.4	0	0
*Alternaria alternaria* f. sp. mali	58.1	30.2	0	0
*Alternaria brassicae* (Berk.) Sacc.	57.4	29.8	2.8	0
*Cercospora arachidicola* Hori	56.8	28.1	2.5	0
*Cladosporium ladosporium*	53.4	26.6	0	0

* inhibition rate; ** positive control chemical.

## Supplementary Materials

The supplementary materials are available online.

## References

[1] János B. (2012). Thoughts and facts about antibiotics: Where we are now and where we are heading. J. Antibiot..

[2] Newman D.J., Cragg G.M., Snader K.M. (2000). The influence of natural products upon drug discovery. Nat. Prod. Rep..

[3] Newman D.J., Cragg G.M. (2007). Natural products as sources of new drugs over the last 25 years. J. Nat. Prod..

[4] Tomihisa O., Shigeru N., Zenji S., Toshio A., Shi-ichi H., Shigo N. (1986). Cyclopropylalanine, an antifungal amino acid of the mushroom *Amanita virgneoides* BAS. Chem. Lett..

[5] Gunter B.K. (2003). Leucine biosynthesis in fungi: Entering metabolism through the back door. Microbiol. Mol. Biol. Rev..

[6] Beltzer J.P., Chang L., Hikkaneen A.E., Kohlhaw G.B. (1986). Structure of yeast Leu4. J. Biol. Chem..

[7] Chanchaem W., Palittapongranpim P. (2002). A variable number of tandem repeats result in polymorphic α-isopropylmalate synthase in *Mycobacterium tuberculosis*. Tuberculosis.

[8] Kraker J.W., Luck K., Textor S. (2007). Two Arabidopsis genes (IPMS1 and IPMS2) encode is α-isopropylmalate synthase, the branchpoint step in the biosynthesis of leucine. Plant Physiol..

[9] Yindeeyoungyron W., Supaporn L.S., Prasit P.P. (2009). Characterization of α-isopropylmalate synthase containing different copy numbers of tandem repeats in *Mycobacterium tuberculosis*. BMC Microbiol..

[10] Konn N., Squire C.J., Baker E.N. (2004). Crystal structure of LeuA from *Mycobacterium tuberculosis*, a key enzyme in leucine biosynthesis. Proc. Natl. Acad. Sci. USA.

[11] Der Carvalho L.P.S., Argyrou A., Blanchard J.S. (2005). Slow-onset feedback inhibition: Inhibition of *Mycobacterium tuberculosis* α-isopropylmalate synthase by leucine. J. Am. Chem. Soc..

[12] Martinez J.P., Sasse F., Brönstrup M., Diez J., Meyerhans A. (2015). Antiviral drug discovery: Broad-spectrum drugs from nature. Nat. Prod. Rep..

[13] Mark S.B., Antony D.B. (2006). Natural products-The future scaffolds for novel antibiotics?. Biochem. Pharmacol..

[14] Dennis C.D., William S.C. (2002). Characterization and toxicity of *Amanita cokeri* extract. J. Chem. Ecol..

[15] Mohana D.C., Raveesha K.A. (2007). Anti-fungal evaluation of some plant extracts against some plant pathogenic field and storage fungi. J. Agric. Technol..

