# Supplementary material for: A Novel Antibiotic Mechanism of l-Cyclopropylalanine Blocking the Biosynthetic Pathway of Essential Amino Acid l-Leucine
Source: Molecules. 2017 Dec 14;22(12):2224. doi: 10.3390/molecules22122224 (PMC6149818; doi:10.3390/molecules22122224)
Supplement: Supplementary file 1 [file molecules-22-02224-s001.pdf]

molecules

MDPI

Article

A Novel Antibiotic Mechanism of L-Cyclopropylalanine Blocking the Biosynthetic Pathway of Essential Amino Acid L-Leucine

Bingji Ma [1,*], Jinwen Shen [2], Wandee Yindeeyoungyeon [3] and Yuan Ruan [1]

1 Department of Traditional Chinese Medicine, Henan Agricultural University, Zhengzhou 450002, China; ruanyuanmbj@163.com
2 Department of Microbiology, Henan Agricultural University, Zhengzhou 450002, China; shenjinwen369@163.com
3 National Center for Genetic Engineering and Biotechnology, NSTDA, Pathum Thani 12120, Thailand; wandee@biotec.or.th
* Correspondence: mbj123@sina.com; Tel.: +86-371-6350-4463

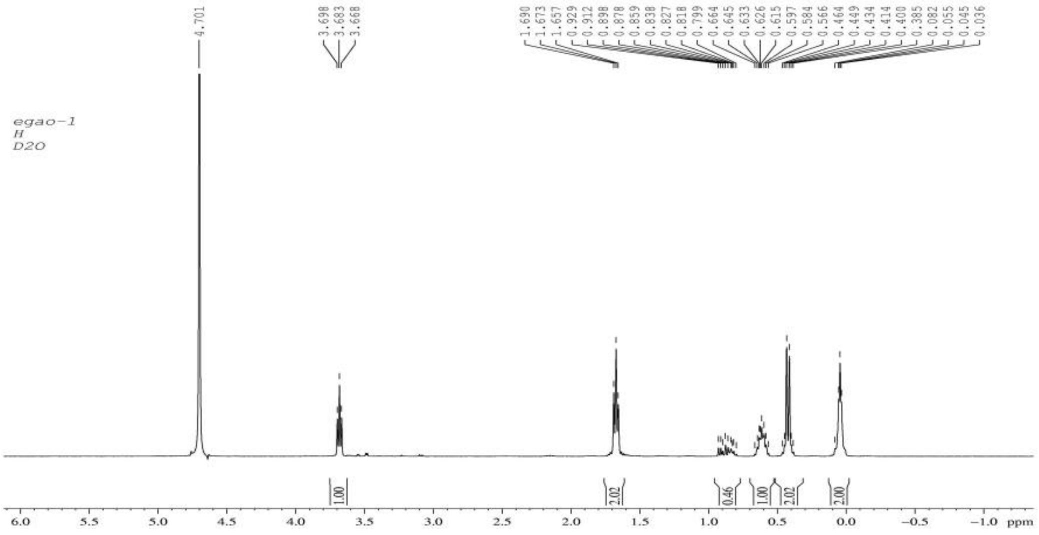

(**a**)

Figure S1. *Cont*.

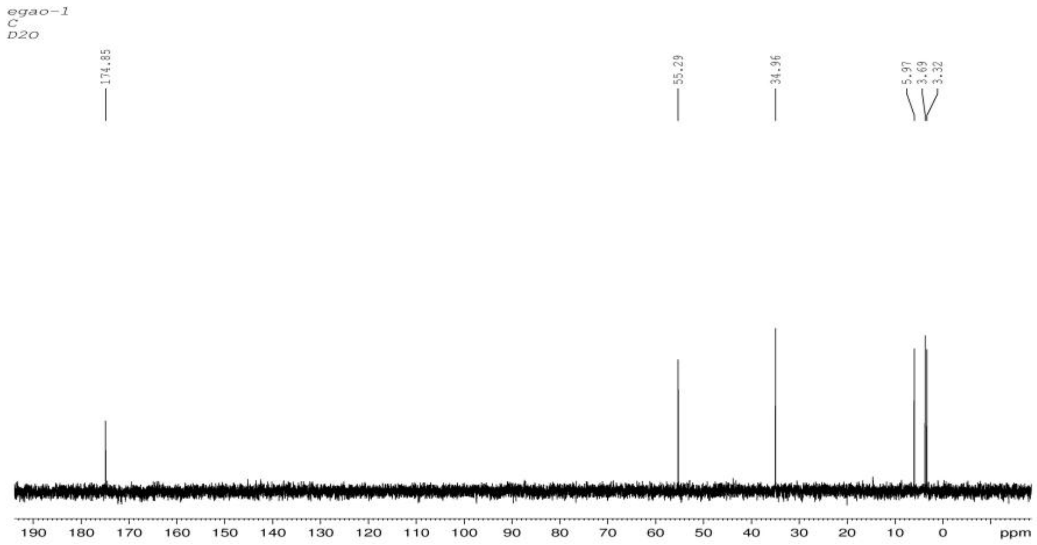

(**b**)

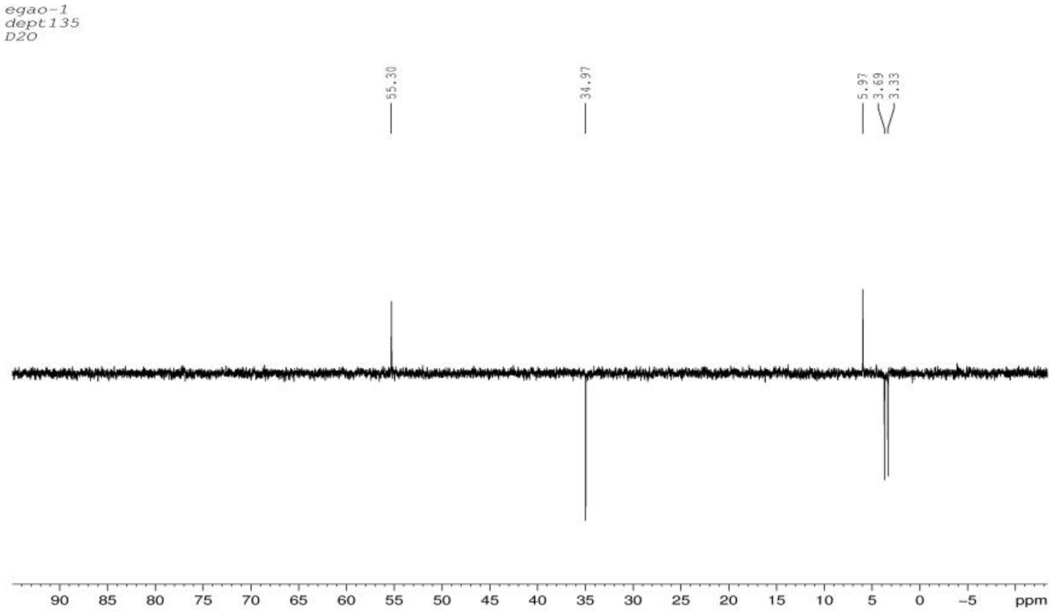

(**c**)

Figure S1. *Cont*.

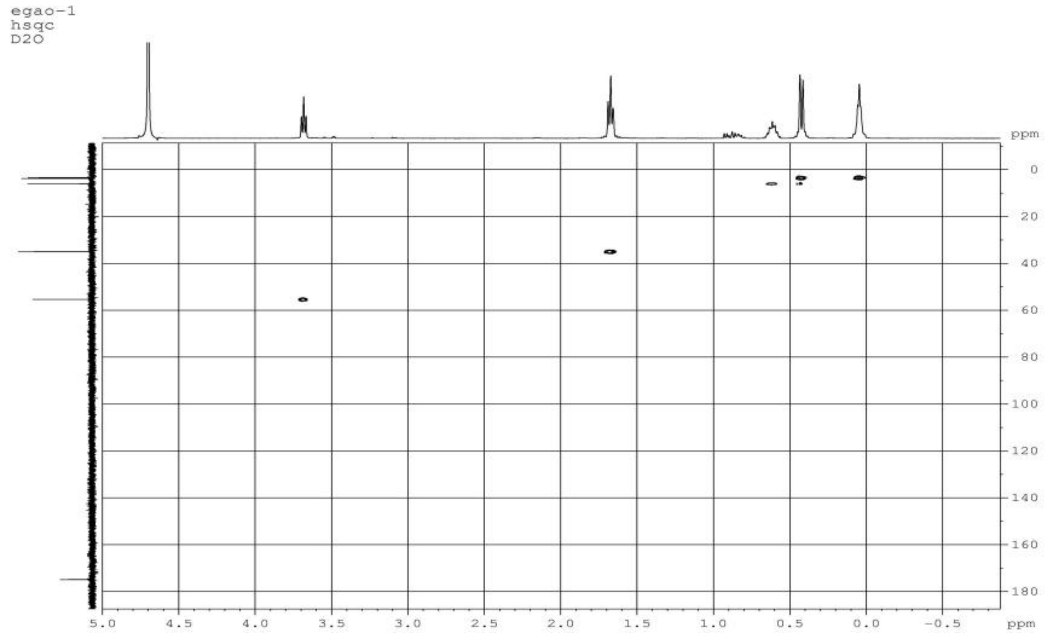

(**d**)

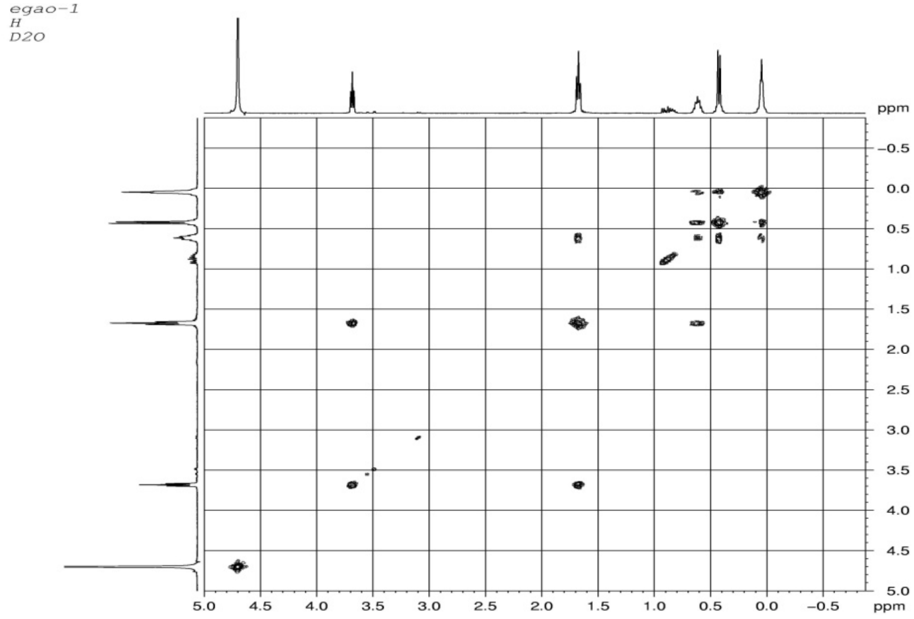

(**e**)

Figure S1. *Cont*.

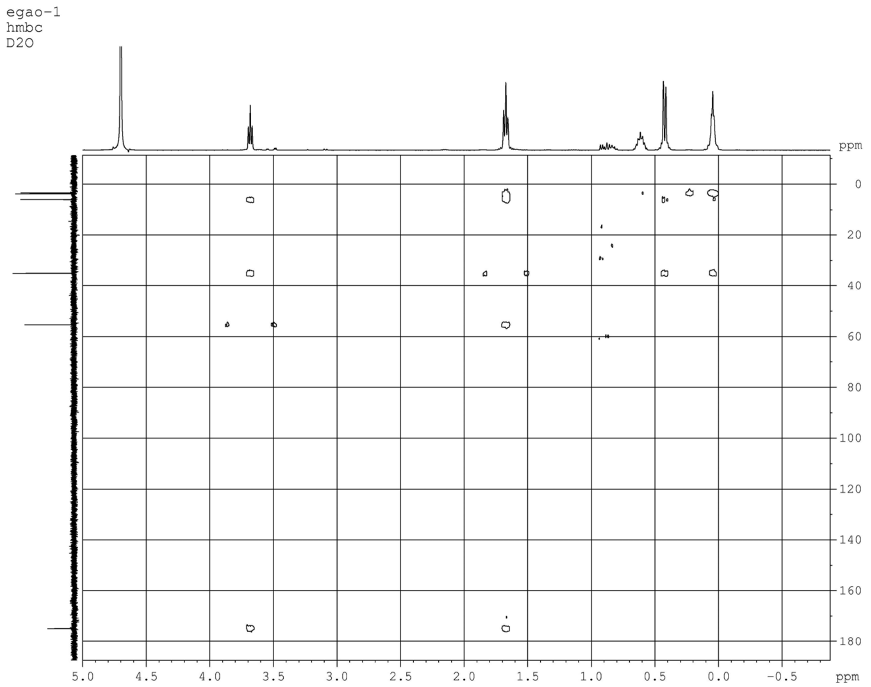

(**f**)

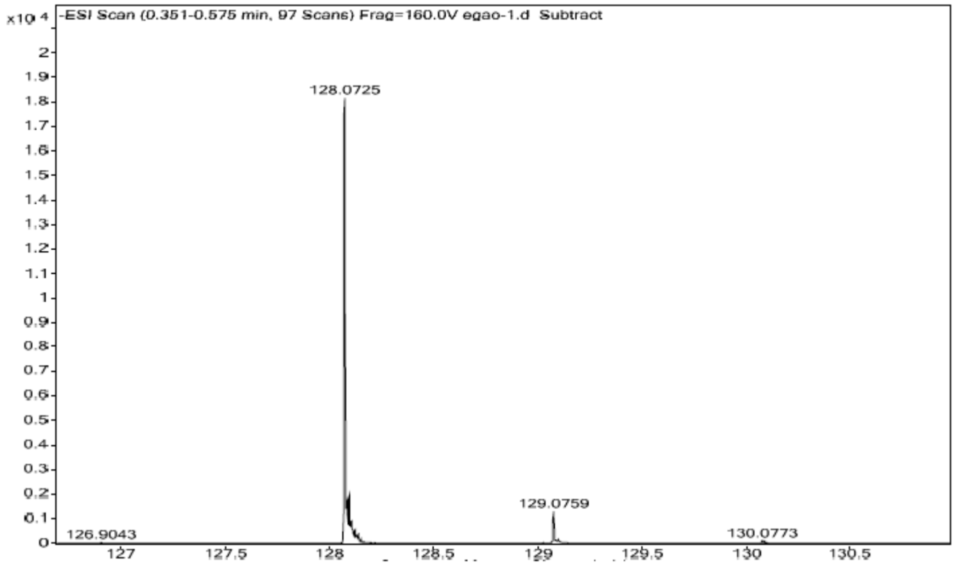

(**g**)

Figure S1. NMR and MS spectral of L-cyclopropylalanine (a: ^{1}H-NMR; b: ^{13}C-NMR; c: DEPT 135; d: HSQC; e: ^{1}H-^{1}H COSY; f: HMBC; g: MS)

Table S1. Inhibitory effects of L-cyclopropylalanine and its derivatives **1**, **2** and **3** on *F. graminearum* Schw

Compounds	IR at 50 μg/mL	IR at 5 μg/mL
L-cyclopropylalanine	77.2	65.5
**1**	62.8	45.5
**2**	9.2	8.8
**3**	5.9	5.0
